# Prompt-Controlled Multi-Modal Tuning for Person-Specific Few-Shot Micro-Expression Recognition

**DOI:** 10.3390/s26196083

**Published:** 2026-09-25

**Authors:** Ruiqi Wang, Kerong Li, Jiateng Liu, Tianxiang Cao, Yingtian Yu, Hengcan Shi, Yaonan Wang

**Affiliations:** 1 School of Artificial Intelligence, The University of Science and Technology Beijing, Beijing 100080, China; wr.maox@hotmail.com; 2 College of Electrical and Information Engineering, Hunan University, Changsha 410082, China; 202304060821@hnu.edu.cn; 3 The Key Laboratory of Child Development and Learning Science of Ministry of Education, Southeast University, Nanjing 211100, China; jiateng_liu@seu.edu.cn; 4 School of Artificial Intelligence and Robotics, Hunan University, Changsha 410082, China; 3139167108@hnu.edu.cn (T.C.); yaonan@hnu.edu.cn (Y.W.); 5 China Mobile Communications Group Hunan, Changsha 410082, China; yuyintian@hn.chinamobile.com

**Keywords:** micro-expression recognition, few-shot, adapter groups, CLIP

## Abstract

Micro-expression recognition (MER) is a challenging step in various multimedia applications, such as media understanding and human–computer interaction, as it can reveal genuine human emotions. However, traditional MER often overlooks person-specific facial nuances, limiting generalization and personalized adaptability in practical applications. In this paper, we propose a novel person-specific few-shot MER benchmark that uses only an image/video of the target person as a reference to extend the boundaries of generalization and personalized adaptability in MER. A Prompt-Controlled Multi-Modal Tuning (PCMMT) framework is presented to tackle this benchmark. We introduce a prompt bottleneck mechanism in PCMMT that leverages text prompts and the CLIP multi-modal embedding space to bridge the query and reference visual inputs. Our prompt bottleneck also controls the flow of information across modalities to extract person-specific, subtle micro-expression motions. Moreover, adapter groups are designed at the layer level, with multi-modal tokens fine-tuned separately to refine person-specific cues and enhance generalization. The experimental results show that the proposed person-specific few-shot setting achieves better personalized generalization than traditional MER settings, and our PCMMT outperforms previous state-of-the-art models across various MER benchmarks.

## 1. Introduction

Micro-expression recognition (MER) is a challenging benchmark in affective computing, aiming to classify subtle, transient facial expressions from video sequences. Micro-expressions (MEs) are brief, involuntary facial movements that can reveal hidden emotions [1,2]. Compared with overt macroscopic expressions, their fleeting, subtle nature makes them difficult to detect, yet they are extremely valuable because they reveal true emotional states. Therefore, they have wide applications in media generation, understanding, and human–computer interaction [3,4].

Recent MER research has shifted from early handcrafted feature-based methods designed to capture local texture changes toward deep learning architectures [5,6]. In particular, transformer-based methods are able to model long-range dependencies among different facial regions and video frames [7,8]. Despite significant advancements in MER technology, two problems still need to be addressed. The first is the small-data problem, where the sample size limits generalization and requires human annotation by psychologists. The second is person-specific expressions, where traditional methods capture only non-generalizable features and fail to dynamically recognize individual differences.

To address these challenges, we propose a novel person-specific few-shot MER benchmark, aiming to identify dynamic, individual-specific MEs using only a single reference sample containing the target individual, as shown in Figure 1. In practical applications such as human–computer interaction and robotics, users can easily provide a photo or a short video as a reference sample. Based on this sample, we can learn a wealth of individual-related information (e.g., facial structures, genders, and ages), which significantly helps us flexibly recognize the user’s MEs. Without this reference, traditional MER can only learn static ME features. Meanwhile, traditional few-shot learning [9,10] primarily aims to increase category diversity. To achieve the task of the proposed new benchmark and make the current MER more practical, we design Prompt-Controlled Multi-Modal Tuning (PCMMT). This method first introduces a prompt bottleneck mechanism that selectively injects personal information from reference samples into the query via textual cues, enabling the model to capture relevant ME cues and avoid information confusion. Furthermore, a set of modality-aware adapters is employed to adapt the frozen CLIP backbone to the MER domain, thereby optimizing personal feature representations and reducing cross-modal information clutter. Extensive experiments validate the effectiveness of the proposed benchmark setting and the PCMMT framework.

## 2. Related Work

### 2.1. Micro-Expression Recognition

MER is inherently more challenging than conventional facial expression analysis due to its extremely short duration (typically less than 0.5 s) and the subtle, low-intensity facial movements involved [11,12]. With the rise of deep learning, data-driven methods have significantly advanced the field by enabling hierarchical feature learning. In particular, architectures such as 3D Convolutional Neural Networks and two-stream networks have been widely adopted to capture spatiotemporal patterns from short video sequences. Meanwhile, Recurrent Neural Networks have been employed to model temporal dependencies by processing sequences derived from frame differences or optical flow representations.

To further address the weak motion signals characteristic of MEs, recent studies [13,14,15,16] have incorporated specialized enhancement strategies. For instance, Eulerian Video Magnification (EVM) has been utilized to amplify subtle facial movements, making them more distinguishable for downstream models. Additionally, facial landmark-guided attention mechanisms have been proposed to focus on key regions of interest. Hybrid approaches have also emerged, combining handcrafted motion descriptors with deep learning features to enhance robustness under low-signal conditions.

### 2.2. Few-Shot Learning

In general, each person can only provide a limited number of samples, usually only a single one. This scenario essentially associates it with Few-Shot Learning, which aims to learn novel concepts based on a few labeled samples. Previous studies mainly attack FSL from the view of meta-learning and metric learning. Methods based on graphs also draw some attention. Meta-learning-based methods focus on learning to learn efficiently based on a few labeled samples. This is to adapt the trained model to different few-shot tasks efficiently based on the support set (the few labeled data). Prototypical Network [17] classifies images based on the prototypes of classes from the support set. Relation Network [18] uses a neural network as the metric to measure the similarity between representations. DeepEMD [19] utilizes a differentiable Earth Moving Distance layer as the metric and trains the model end-to-end. There are also image-retrieval-based studies [20] and reinforcement learning-based ones [21].

However, we argue that the variety of people are the vital challenge for MER. Inspired by the methodology of prototypical learning, we introduce a person-specific few-shot MER benchmark. Instead of using global emotion centroids, we treat each subject’s reference as a person-specific prototype. This reference serves as a baseline for calibrating the query features, enabling the model to focus on relative changes rather than absolute feature positions in the embedding space.

### 2.3. Vision-Language Large Models

The advent of large-scale Vision-Language Models (VLMs), exemplified by CLIP [22], has revolutionized representation learning. By aligning visual and textual modalities in a shared embedding space, these models provide robust and semantically rich priors that generalize well to downstream tasks, even under zero-shot or few-shot settings. However, directly applying such large-scale models to fine-grained tasks like MER remains challenging. Full fine-tuning is often computationally expensive and prone to overfitting due to the limited availability of MER data. As a result, Parameter-Efficient Fine-Tuning (PEFT) strategies, including adapters, prompt tuning, and LoRA, have become essential. These approaches enable effective domain adaptation by optimizing only a small number of additional parameters while keeping the pretrained backbone frozen, thereby preserving general knowledge while adapting to task-specific nuances.

## 3. Person-Specific Few-Shot Micro-Expression Recognition Benchmark

### 3.1. Person-Specific Few-Shot Setting

An *N*-way *K*-shot task contains a support set S containing *K* labeled samples for each of the *N* classes and a query set Q containing unlabeled samples for evaluation. Given a query sample *s*, the few-shot learning model $f_{\theta }\left(\cdot \right)$ predicts the label of a query sample based on the support set:

(1) $$\hat{y}=f_{\theta }\left(s,S\right),\;s\in Q.$$

where $\hat{y}$ denotes the estimated classification probability. In prototypical learning, each class is represented by a prototype, and classification is performed according to the distances between query features and class prototypes.

In contrast, in our person-specific few-shot setting, only one reference sample is provided to define the person, and various ME classes of this person shall be recognized. Let $D=\{\left(s_{i}^{j},y_{i}^{j},r_{i}\right)\}$ denote the ME dataset, where $s_{i}^{j}$ is the *j*-th sample of the *i*-th person (subject), $y_{i}^{j}$ is the MER label of this sample, and $r_{i}$ represents the person identity. With data reorganized as above, we can define our person-specific few-shot benchmark as follows:

(2) $$\hat{y}=f_{\theta }\left(s_{i}^{j},r_{i}\right)$$

where $s_{i}^{j}$ is the *j*-th query sample of the *i*-th person that is recognized to an ME class. $r_{i}$ is the reference sample that defines the *i*-th person.

### 3.2. Dataset Processing

We can easily leverage traditional MER datasets to build the benchmark of our person-specific few-shot MER. A traditional MER dataset usually involves diverse subjects. For each subject, there are multiple samples ranging in various classes. Meanwhile, the dataset is divided into the training and testing sets in accordance with subjects; namely, there is no person overlap between the two sets.

Based on traditional datasets, we select a sample for each person as the reference. Our benchmark can take any ME as the sample. Normally, we recommend neutral expressions as the samples. Compared to using other expression samples as references, neutral faces are more practical and easier to acquire in real-world scenarios (e.g., identity photos or videos), making the proposed setting more applicable to real-world deployments. Specifically, for each person, we first randomly extract a sample video from traditional MER datasets. Then, we designate the frames without any ME motion as neutral expressions, as illustrated in Figure 2. Furthermore, we ensure that the references and queries that we select do not come from the same sample video, thereby preventing data leakage.

### 3.3. Person-Specific Few-Shot vs. Normal Few-Shot

Traditional few-shot learning provides new category cues during inference, aiming to extend the generalization ability of recognizing new categories. This setting is widely used in object recognition because there are diverse object categories in the real world. However, as defined by the psychologists, MEs contain several categories. These categories are easily covered in the training set, and thus, providing category cues during inference cannot significantly enhance the generalization ability.

In contrast, the diversity of humans is a vital challenge for MER. People of various ages, genders, and cultures express their emotions in different ways. Moreover, different people also have their own facial appearances. Therefore, we propose the person-specific few-shot benchmark to generalize MER for diverse people by only adding a reference sample during inference. Our benchmark focuses on cross-subject generalization by leveraging a person-specific reference sample, which enables the model to adapt to any individual. Therefore, there will be no age, gender, or cultural biases [23].

## 4. Methods

### 4.1. Prompt-Controlled Multi-Modal Tuning

As shown in Figure 3, PCMMT consists of four main steps: (1) a data preprocessing step including a CLIP visual tokenizer and a CLIP textual encoder to transform the inputs into a unified token sequence; (2) a CLIP visual encoder to extract features and determine the category; (3) a prompt bottleneck module to control the information flow to capture person-specific ME features; (4) adapter groups enabling parameter-efficient fine-tuning to refine person-specific tokens.

The inputs of PCMMT are a query sample *s*, a reference sample *r* defining the person, and a set of predefined textual prompts {P}. We expect PCMMT to predict an ME category for the query sample. Because CLIP only accepts image inputs, rank pooling [24] is employed to transform query and reference videos into dynamic images while preserving temporal motion information. Given a query video sequence *s* and a reference video sequence *r*, the corresponding dynamic images are obtained by the following:

(3) $$\begin{array}{cc} s^{\prime } & =RP(s), \end{array}$$

(4) $$\begin{array}{cc} r^{\prime } & =RP(r), \end{array}$$

where RP(·) denotes the rank pooling operation.

The resulting images are tokenized by the CLIP visual tokenizer VT to produce $x_{0}^{q}$ and $x_{0}^{ref}$:

(5) $$\begin{array}{cc} x_{0}^{q} & =\mathrm{VT}(s^{\prime }), \end{array}$$

(6) $$\begin{array}{cc} x_{0}^{ref} & =\mathrm{VT}(r^{\prime }). \end{array}$$

We set the descriptions of key MER elements (e.g., “motion”, “eye”, and “mouth”) as our textual prompts {P}. The CLIP textual encoder TE embeds the prompts {P}:

(7) $$\begin{array}{cc} x_{0}^{p} & =\mathrm{TE}(\{P\}) \end{array}$$

where $x_{0}^{p}$ is the cascading tokens embedded for each prompt.

To align the prompt tokens with the visual feature space, a fully connected layer is employed to project $x_{0}^{p}$ into the same embedding space as $x_{0}^{q}$ and $x_{0}^{ref}$:

(8) $$x_{0}^{p}=x_{0}^{p}W_{0},$$

where $W_{0}$ denotes the learnable projection matrix. $x_{0}^{p}$ shares the same feature dimension as the query and reference tokens, enabling subsequent cross-modal interaction.

Then, a learnable classification token (cls token) $x_{0}^{cls}$ is defined directly in the token space. We concatenate all the above token embeddings as the input of our visual encoder:

(9) $$x_{0}=\left[x_{0}^{ref},x_{0}^{p},x_{0}^{q},x_{0}^{cls}\right].$$

The input is fed into the CLIP ViT visual encoder VE with adapter groups adp as well as a prompt bottleneck (mainly defined by an attention mask M) to extract the person-specific information and embed this information into $x_{0}^{cls}$:

(10) $$x_{L}=\mathrm{VE}\left(x_{0}|M\right).$$

Finally, a classification head is applied to the final classification token $x_{L}^{cls}$ to predict the ME label $\hat{y}$:

(11) $$\hat{y}=\mathrm{class}\left(x_{L}^{cls}\right),$$

where class(·) denotes the classification head.

The network is optimized using the cross-entropy (CE) loss:

(12) $$L=\mathrm{CE}(\hat{y},y_{g}),$$

where $\hat{y}$ denotes the predicted class probability produced by the classification head, $y_{g}$ is the corresponding ground-truth label, and CE(·) denotes the cross-entropy loss function. The network is trained by minimizing the loss L.

### 4.2. Prompt Bottleneck

By controlling the information flow from the reference to the textual prompts and then from the textual prompts to the query, the textual prompts act as an information bottleneck that filters reference information before it is delivered to the query, preventing direct information flow between the reference and query. Our prompt bottleneck has two advantages: (1) extracting person-specific MER information and (2) ensuring that the reference embedding tokens are not contaminated by extraneous information. The bottleneck here is designed to regulate information flow rather than reduce feature dimensionality [25,26].

MER-specific prompts guide the retrieval of relevant visual cues in CLIP’s shared embedding space (e.g., “motion”, “eye”, and “mouth”), while an attention mask preserves reliable person-specific information by preventing the contamination of reference tokens. We define the input tokens of the *l*-th transformer block as follows:

(13) $$x_{l-1}=\left[x_{l-1}^{ref},x_{l-1}^{p},x_{l-1}^{q},x_{l-1}^{cls}\right],$$

Prompt tokens are designed to retrieve relevant information from the reference and subsequently guide the query features, while reference tokens remain uncontaminated. Thus, the prompts serve as an information bottleneck between the reference and query representations. Formally, given the input sequence of *D* tokens, we define the attention mask for the *h*-th attention head of the transformer block as follows:

(14) $$M_{h}={\left[m_{ab}\right]}^{D\times D},$$

where ab notes that the *a*-th token gathers information from the *b*-th token.

With the designed information flow, the prompt bottleneck attention module can be formulated as follows:

(15) $$m_{ab}=\left\{\begin{array}{cc} -\infty , & a\in i^{ref}\;\mathrm{and}\;b\in i^{q}\cup i^{cls},\\ -\infty , & a\in i^{q}\;\mathrm{and}\;b\in i^{ref},\\ 0, & \mathrm{otherwise}, \end{array}\right.$$

where $i^{ref}$, $i^{q}$, $i^{p}$, and $i^{cls}$ denote the indices of reference tokens $x_{l}^{ref}$, query tokens $x_{l}^{q}$, and classification token $x_{l}^{cls}$, respectively. Following the common practice of masked attention, the same mask is applied to the attention scores of each attention head before the softmax operation:

(16) $${\mathrm{Attn}}_{h}=\mathrm{Softmax}\left(\frac{Q_{h}K_{h}^{⊤}+M_{h}}{\sqrt{d_{h}}}\right),$$

where $Q_{h}$, $K_{h}\in R^{D\times d_{h}}$ denote the query and key matrices of the *h*-th attention head, respectively. $d_{h}$ is the feature dimension of each attention head, and ${\mathrm{Attn}}_{h}\in R^{D\times D}$ denotes the resulting attention weight matrix.

The attention output of the *h*-th attention head is obtained by multiplying the attention weights with the corresponding value matrix. The outputs from all attention heads are concatenated and linearly projected, followed by a residual connection and layer normalization:

(17) $$\begin{array}{cc} O_{h} & ={\mathrm{Attn}}_{h}V_{h}, \end{array}$$

(18) $$\begin{array}{cc} O_{l} & =\mathrm{Concat}\left(O_{1},\ldots ,O_{H}\right)W_{l}^{out}, \end{array}$$

(19) $$\begin{array}{cc} Z_{l} & =\mathrm{LN}(x_{l-1}+O_{l}), \end{array}$$

where $V_{h}\in R^{D\times d_{h}}$ is the value matrix of the *h*-th attention head, where *H* denotes the number of attention heads, $W_{l}^{out}\in R^{d\times d}$ denotes the output projection matrix, $x_{l-1}$ is the input tokens of the *l*-th attention block, and LN(·) denotes the layer normalization operation.

The normalized features $Z_{l}$ are subsequently processed by the feed-forward network (FFN):

(20) $$F_{l}=\mathrm{FFN}\left(Z_{l}\right).$$

To enable parameter-efficient adaptation, a lightweight module named adapter groups is inserted after the feed-forward network (FFN):

(21) $$A_{l}=\mathrm{adp}\left(F_{l}\right),$$

where $F_{l}$ denotes the output feature of the FFN at the *l*-th transformer block, adp(·) denotes the adapter group, and $A_{l}$ represents the adapted feature produced by the adapter groups.

Finally, the output of the *l*-th transformer block is obtained through a residual connection followed by layer normalization:

(22) $$x_{l}=\mathrm{LN}\left(Z_{l}+A_{l}\right),$$

where $x_{l}$ denotes the output tokens of the *l*-th transformer block.

### 4.3. Adapter Groups

We adopt adapter groups to enable the parameter-efficient fine-tuning of the pretrained CLIP. Following [27], lightweight adapters are inserted into the frozen CLIP visual encoder via residual connections. Only the adapter parameters are optimized, enabling parameter-efficient adaptation to the MER domain. We design our adapters as two-layer MLPs (multilayer perceptrons). Formally, given an input token $x_{l-1}^{\prime }\in R^{d_{l}}$, an adapter can be formulated as follows:

(23) $$\mathrm{adp}\left(x_{l-1}^{\prime }\right)=W_{l}^{up}\times \mathrm{ReLU}\left(W_{l}^{down}\times x_{l-1}^{\prime }\right)+x_{l-1}^{\prime },$$

where $W_{l}^{down}\in R^{k\times d_{l}}$ and $W_{l}^{up}\in R^{d_{l}\times k}$ denote the learnable downsample and upsample matrices, respectively. $d_{l}$ denotes the feature (embedding) dimension of the input tokens in the *l*-th transformer block. *k* is the hidden dimension of the adapter, and ReLU is the activation function.

As our token sequence $\left[x_{l-1}^{\prime ref},x_{l-1}^{\prime p},x_{l-1}^{\prime q},x_{l-1}^{\prime cls}\right]$ is derived from diverse modalities, we use different adapters for different token types. For $x_{l-1}^{\prime ref}$, $x_{l-1}^{\prime p}$, and $x_{l}^{\prime q}$, we select the same adapter (vision) for $x_{l-1}^{\prime ref}$ and $x_{l-1}^{\prime q}$, and then we select a different adapter (text) for $x_{l-1}^{\prime p}$. For the classification token $x_{l-1}^{\prime cls}$, we do not use any adapter because the token can be directly optimized.

## 5. Experiment

### 5.1. Datasets and Metrics

We evaluate our model on widely used ME datasets: SMIC [12] and SAMM [28]. The SMIC dataset [12] involves 164 samples, 16 subjects/people, and three categories: *Positive*, *Negative*, and *Surprise*. There are 159 samples and 29 subjects/people in the SAMM dataset [28]. It includes five categories: *Anger*, *Contempt*, *Happiness*, *Surprise*, and *Others*. The evaluation protocols align with common MER community standards [29,30]. We adopt a subject-independent LOSO protocol, ensuring that no subject appears in both the training and testing sets. Our validation also follows the subject-independent principle. The reference samples are selected only after the held-out subject has been determined. The evaluation metrics are the classification accuracy and F1-score, reported as mean ± standard deviation.

### 5.2. Implementation Details

Pretrained CLIP-ViT-B/16 is adopted as the visual large model with the corresponding text encoder used to extract text embeddings. Adapters are designed as two-layer MLPs with 512 hidden units and a residual path. Two different adapters are leveraged for vision tokens and text tokens, respectively. We argue that the classification tokens do not need an adapter because they are learnable tokens. By default, three words are used as textual prompts, namely “motion”, “eye”, and “mouth”. The model is optimized using AdamW [31], with the initial learning rates $1\times 10^{-4}$ for adapters, and $5\times 10^{-5}$ for prompts. The cosine annealing scheduler is leveraged for a total of 500 epochs, where the learning rates reach $1\times 10^{-6}$ at the final epoch for all optimizable parameters. The batch size is set to 16, with a weight decay of $1\times 10^{-4}$. The random seed is fixed to 42. All facial images are preprocessed without augmentation and handling of class imbalance, cropped to [224 × 224], and normalized using the mean and standard deviation of the CLIP pretraining data. Model selection is based on the highest accuracy, and the corresponding checkpoint was used for testing. The proposed model contains 0.005M trainable parameters. All experiments are performed on an NVIDIA 4090 GPU.

### 5.3. Main Results

In comparison with previous MER methods mentioned in Table 1 and Table 2, we adopt four representative methods, namely Off- ApexNet [32], I3D-MOCO [33], SRMCL [34], and LTR3O [35], for the proposed person-specific few-shot setting by integrating with prototype learning. Specifically, we leverage prototypical networks [17] with necessary changes. The experimental results are reported in Table 1 and Table 2 for the SMIC dataset and SAMM dataset, respectively, where the gray background notes experiments under the person-specific few-shot setting.

The proposed person-specific few-shot setting boosts MER. In Table 1 and Table 2, both Off-ApexNet and SRMCL under person-specific few-shot settings report an increased accuracy and F1-score compared with their naive counterparts. These results validate that the proposed person-specific few-shot setting extends the ability for MER.

The proposed PCMMT performs better than naive integration with prototypical learning. Compared with approaches under the person-specific few-shot setting, our PCMMT method achieves superior performance. On the SMIC dataset, our PCMMT outperforms SRMCL+Proto by 7.92% in accuracy. On the SAMM dataset, PCMMT yields 5.88% improvements under the measure of accuracy. PCMMT also exhibits smaller variations. On SMIC, PCMMT achieves a standard deviation of 0.89%, which is lower than those of the other methods under the person-specific setting. Similarly, on SAMM, PCMMT achieves a standard deviation of 1.61%. The relatively small standard deviations and narrow confidence intervals indicate that PCMMT provides more stable performance. Our PCMMT method outperforms previous state-of-the-art methods in MER. On SMIC, the proposed PCMMT outperforms previous state-of-the-art methods, LTR3O, by 6.09% in accuracy. On SAMM, our PCMMT achieves 0.044 gains under the F1-score measure.

### 5.4. Ablation Study

We further conduct ablation studies from various perspectives to validate the effectiveness of each design in our PCMMT.

#### 5.4.1. Design of Prompt Bottleneck

First, we investigate the effect of the prompt bottleneck from two complementary aspects: the presence of prompt tokens and the introduction of the bottleneck attention mechanism. To provide a comprehensive analysis, we design four experimental settings corresponding to all possible combinations based on the CLIP backbone: (1) without prompts and without the bottleneck, (2) with prompts only, (3) with the bottleneck only, and (4) with both prompts and the bottleneck. Readers can refer to Figure 3 for better understanding. Here, “prompt” indicates the introduction of textual prompts and “bottleneck” implies the control of the information flow through which tokens of the reference sample gather non-information from others. We also compare the performance of VIT and CLIP as backbones, demonstrating the effectiveness of CLIP as the backbone.

The results, summarized in Table 3, allow us to isolate the contribution of each component. One can see that the designs of the prompt and bottleneck both contribute to the final performance by 0.8% in accuracy, respectively. When combined together, a profit of 3% in accuracy can be achieved, which is much better than that for the independent ones. The results not only validate the effectiveness of our prompt and bottleneck but also confirm the unified design of our prompt bottleneck module. This is reasonable because prompts alone cannot solve the problem of a contaminated reference in forward propagation, and the bottleneck alone cannot select relevant information from a reference. With the unified prompt bottleneck module, relevant information in the reference can be retrieved with prompt guidance without contamination of the reference.

#### 5.4.2. Design of Adapter Groups

We analyze the different designs of adapter groups. There are two main questions to answer. The first is the shareability of adapters. This explains the necessity of adopting different adapters for different groups of tokens and finds the possible design of a shared adapter for more tokens. The second is the existence of adapters. We argue that some adapters are not necessary, especially for the classification tokens, which are learnable themselves. The experimental results are shown in Table 4, which demonstrate the effectiveness of our design that uses different adapters for multi-modal tokens.

#### 5.4.3. Prompt Choices

Because the textual prompts are predefined, their specific formulation can significantly influence model performance. To evaluate this effect, we conduct ablation studies on different groups of prompts, as summarized in Table 5. In particular, we consider three representative settings: (1) MER-specific prompts, including “motion”, “eye”, and “mouth”, which explicitly target subtle facial movements; (2) a mixed and more general set, such as “motion” and “vision”; and (3) region-focused but less motion-aware prompts, including “face”, “eye”, and “mouth”. As shown in Table 5, MER-specific prompts report leading performance. General prompts and region-focused prompts lack descriptions of MEs and thus may fail to guide the retrieval of corresponding information from the reference.

#### 5.4.4. Comparison of Confidence Intervals

As shown in Table 6, PCMMT exhibits narrower 95% confidence intervals than the other methods under the person-specific setting. Specifically, PCMMT achieves confidence intervals of [86.72%, 87.66%] on SMIC and [80.98%, 82.26%] on SAMM, corresponding to interval widths of 0.94% and 1.28%, respectively. The narrower confidence intervals indicate lower variation across evaluation folds and suggest that the performance of PCMMT is more stable under different subjects.

#### 5.4.5. Visualization Results

We visualize the prediction confidence scores of different variants on the SMIC dataset, as shown in Figure 4. The prediction confidence scores of the ground-truth categories gradually increase with the introduction of each component. Compared with the baseline, the introduction of the prompt bottleneck and adapter groups improves the confidence scores of the correct classes, while the complete PCMMT achieves the highest prediction confidence scores (e.g., increasing from 0.518 for the baseline to 0.880 for PCMMT). This demonstrates that the proposed components effectively enhance the model’s ability to capture person-specific information and learn more discriminative ME representations.

Furthermore, visualizations of the feature heatmaps in Figure 5 provide an analysis of the learned activation regions. Compared with the baseline and individual components, PCMMT focuses more accurately on discriminative facial regions related to micro-expression changes. This demonstrates that the prompt bottleneck and adapter groups facilitate more effective information interaction and parameter-efficient adaptation, leading to improved person-specific feature extraction.

## 6. Conclusions

In this paper, we present a person-specific few-shot MER benchmark and prompt-controlled multi-modal tuning (PCMMT). Our person-specific few-shot setting can expand the boundaries of MER by easily adding a reference sample, which benefits many real-world applications, such as multimedia generation, understanding, human–computer interaction, and service robots. To complete this task, our PCMMT creatively designs the prompt bottleneck and adapter groups, which have been proven to be effective. The prompt bottleneck leverages text to bridge visual queries and references and can control the information flow to extract person-specific features. Meanwhile, the adapter groups can fine-tune the multi-modal parameters effectively and ensure computational efficiency. Experiments demonstrate the effectiveness of both our person-specific few-shot MER and the PCMMT method. In the future, we will design more accurate frameworks to solve MER problems in the real world.

## Figures and Tables

**Figure 1:**
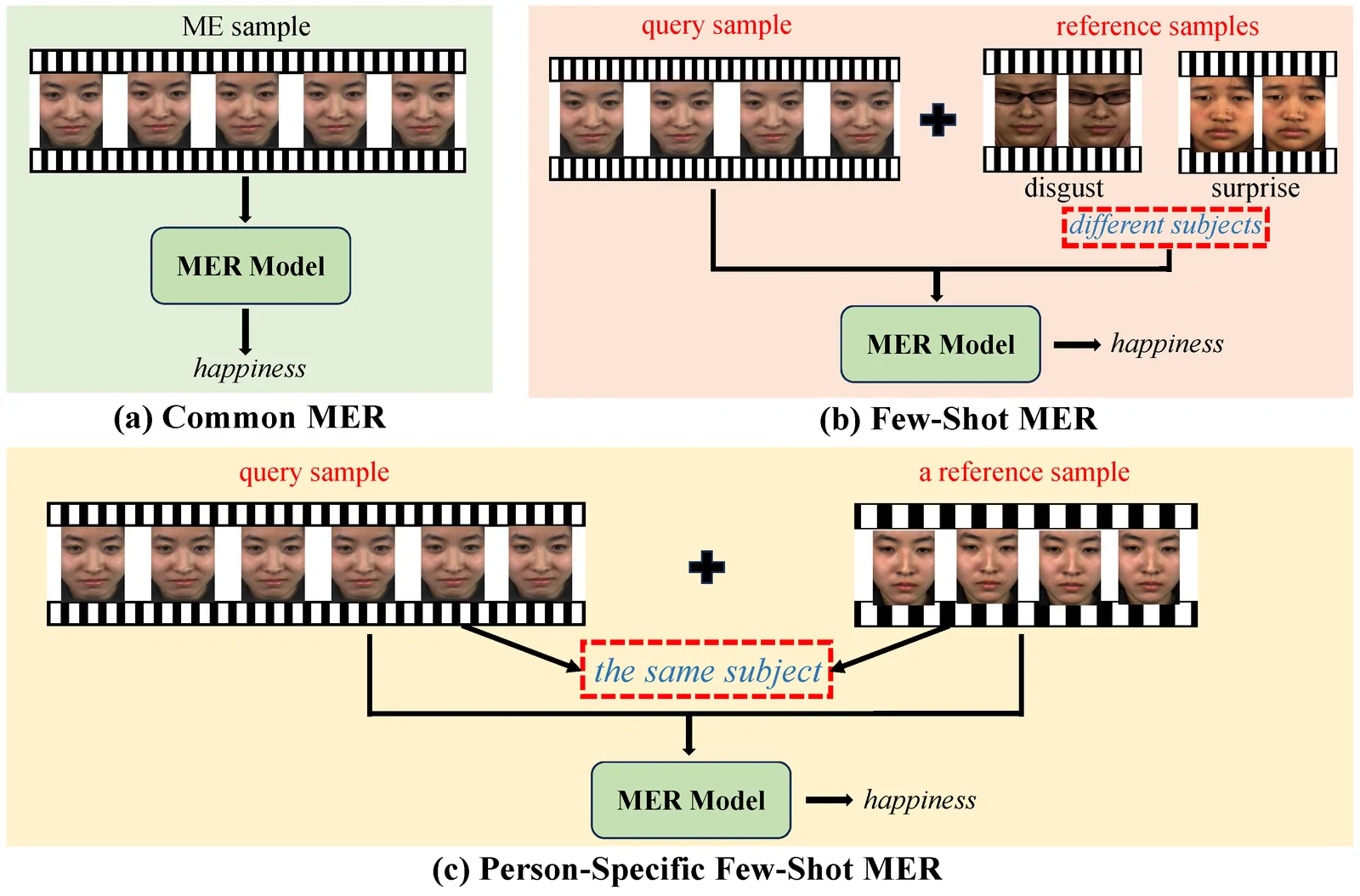
(**a**) The traditional MER benchmark. (**b**) The normal few-shot MER benchmark. (**c**) Our person-specific few-shot MER benchmark.

**Figure 2:**
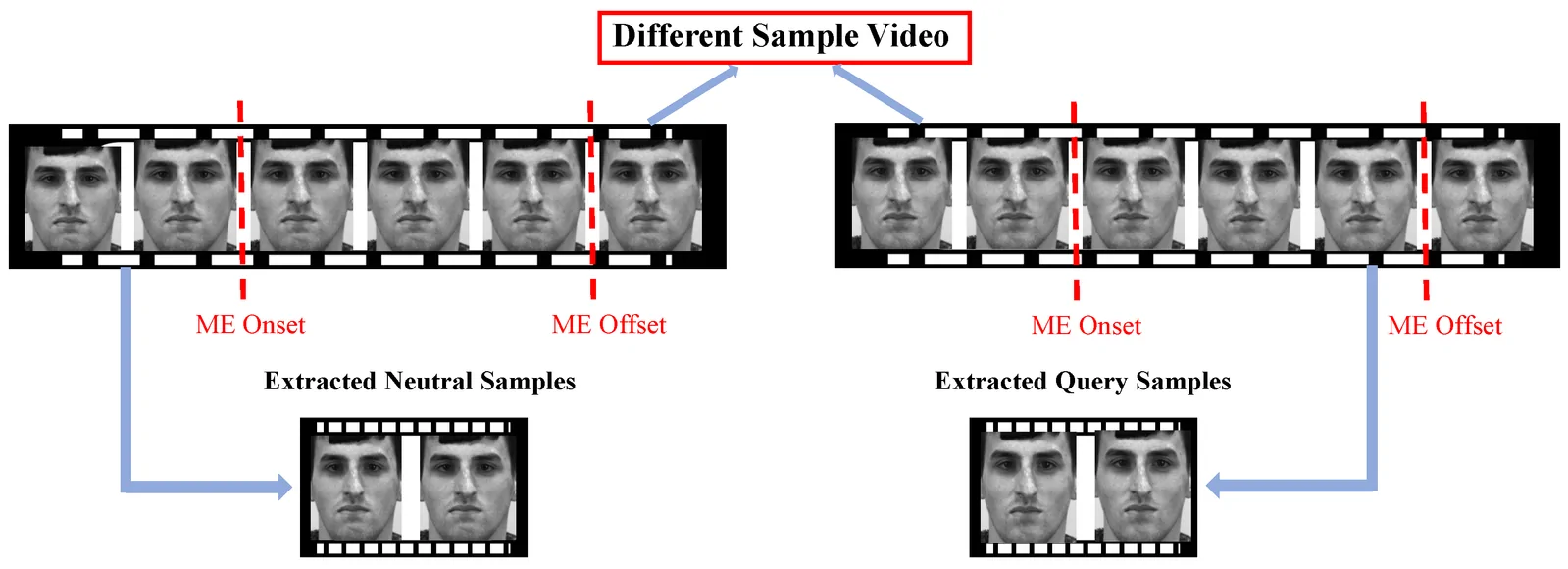
Extraction process of the neutral samples and query samples. The neutral samples are extracted from the frames that are not in the range [ME Onset, ME Offset].

**Figure 3:**
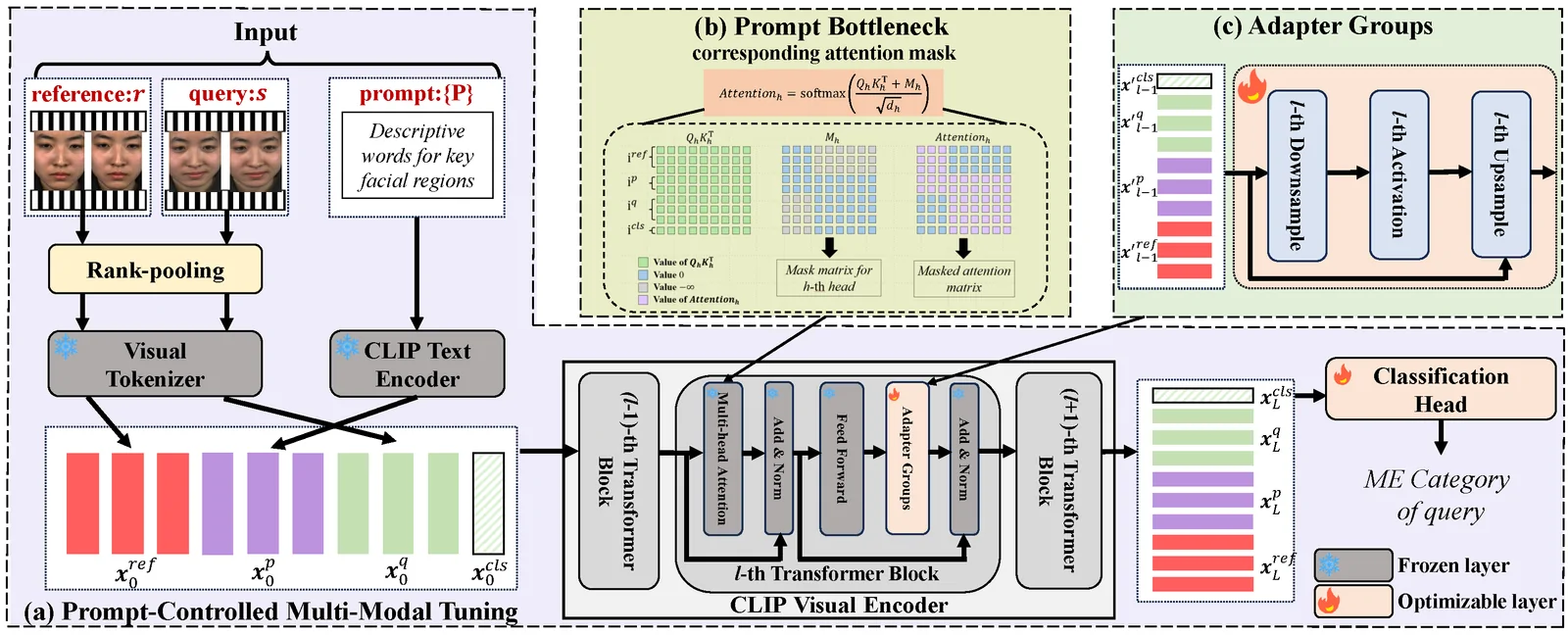
(**a**) Illustration of the proposed **PCMMT** for person-specific few-shot MER. (**b**) The proposed **Prompt Bottleneck** module. (**c**) The proposed **Adapter Groups** module.

**Figure 4:**
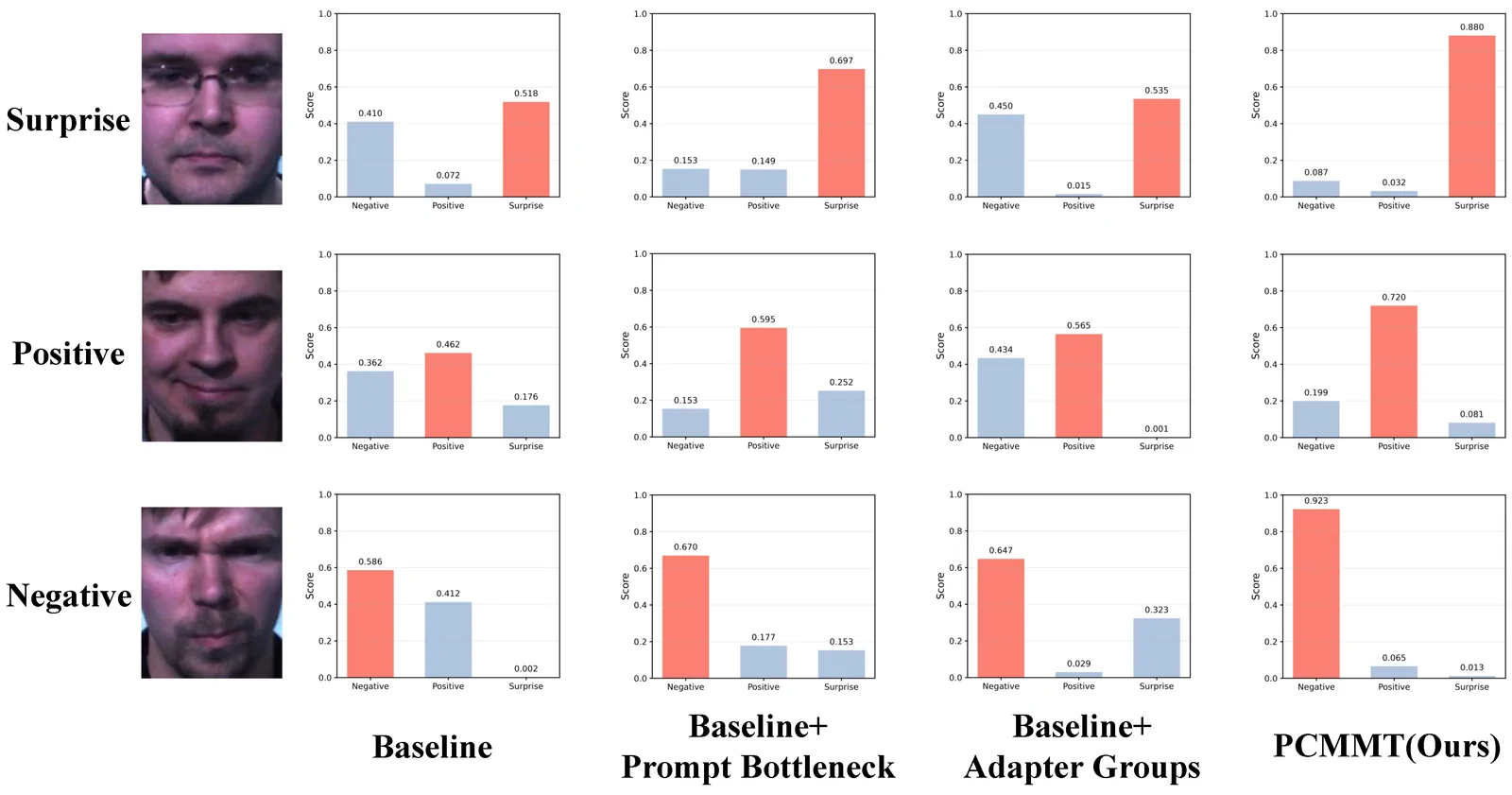
Comparison of prediction confidence scores of different methods and ablation settings on samples from the SMIC dataset. From top to bottom, the samples correspond to surprise, positive, and negative categories.

**Figure 5:**
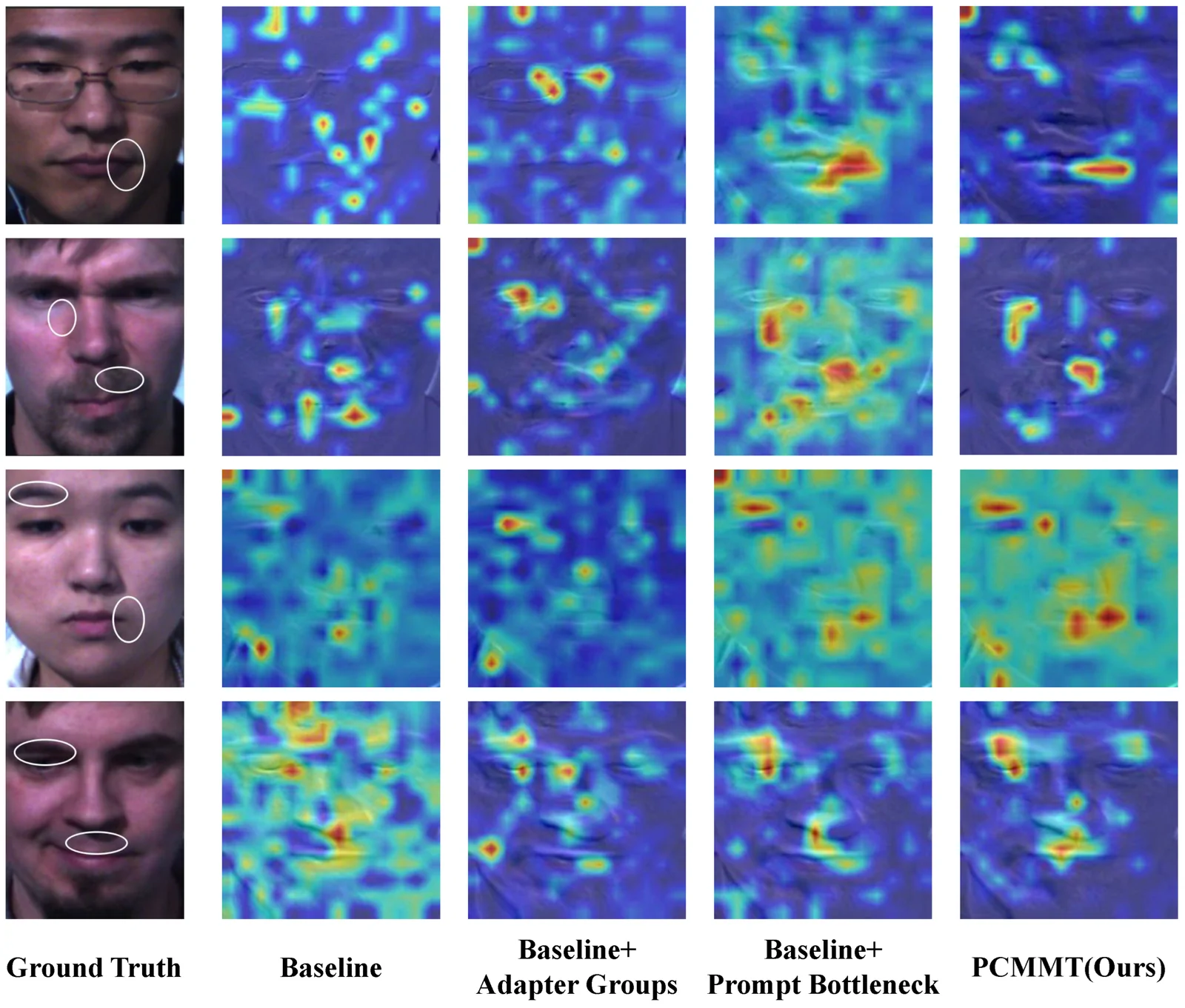
Grad-CAM visualization of different ablation settings on the SMIC dataset. PCMMT achieves more precise attention localization compared with other groups.

**Table 1:** Comparison between the proposed method and previous work on SMIC. Gray background notes approaches under the proposed person-specific few-shot setting.

Method	Accuracy	F1-Score
DSSN [36]	63.41%	0.6461
Off-ApexNet [32]	67.68%	0.6709
KD [37]	76.10%	0.7100
KFC [38]	65.85%	0.6638
SLSTT [39]	73.71%	0.7240
I3D-MOCO [33]	75.61%	0.7492
LVTEA [40]	82.03%	0.8137
SRMCL [34]	78.98±1.25%	0.7887±0.0219
LTR3O [35]	81.10±1.28%	0.8132±0.0199
Off-ApexNet+proto	74.39±1.30%	0.6958±0.0212
I3D-MOCO+proto	76.83±1.24%	0.7632±0.0173
SRMCL+proto	79.27±1.12%	0.7845±0.0245
LTR3O+proto	82.93±1.04%	0.8052±0.0175
**PCMMT (Ours)**	87.19±0.89%	0.8212±0.0147

**Table 2:** Comparison between the proposed method and previous work on SAMM. Gray background notes approaches under the proposed person-specific few-shot setting.

Method	Accuracy	F1-Score
DSSN [36]	57.35%	0.4644
Off-ApexNet [32]	68.18%	0.5423
Graph-TCN [41]	75.00%	0.6985
KFC [38]	63.24%	0.5709
SLSTT [39]	72.39%	0.6400
I3D-MOCO [33]	68.38%	0.5436
MMNet [42]	77.89%	0.7552
SRMCL [34]	74.63±1.81%	0.6599±0.0322
LTR3O [35]	80.15±1.76%	0.7574±0.0266
Off-ApexNet+proto	72.06±1.98%	0.6359±0.0312
I3D-MOCO+proto	71.32±1.63%	0.6640±0.0327
SRMCL+proto	75.00±1.78%	0.6719±0.0233
LTR3O+proto	80.88±1.72%	0.7654±0.0231
**PCMMT (Ours)**	81.62±1.61%	0.8125±0.0224

**Table 3:** Results for different designs of prompt bottleneck on SMIC.

Backbone	Prompt	Bottleneck	Accuracy	F1-Score
CLIP-VIT-B/16	**✓**	**✓**	**87.19%**	**0.8212**
CLIP-VIT-B/16	**✓**	**✗**	85.37%	0.6596
CLIP-VIT-B/16	**✗**	**✓**	85.37%	0.7185
CLIP-VIT-B/16	**✗**	**✗**	84.15%	0.6501
VIT-B/16	**✗**	**✗**	77.44%	0.7176

**Table 4:** Results for different adapter groups on SMIC. Vision means the query and reference samples. “-” indicates no adapter. “c”, “v”, and “t” denote the adapters for the cls, vision, and text modalities, respectively. “vt” is a joint adapter for both vision and text modalities, while “cvt” means an adapter for all three modalities.

CLS	Vision	Text	Accuracy	F1-Score
c	v	t	84.76%	0.7814
-	v	t	**87.19%**	**0.8212**
-	vt	vt	85.98%	0.7814
cvt	cvt	cvt	85.37%	0.7185

**Table 5:** Results for different prompt choices on the SMIC dataset.

Prompt	Accuracy	F1-Score
“motion”, “eye”, “mouth”	**87.19%**	**0.8212**
“motion”, “vision”	85.96%	0.6613
“face”, “eye”, “mouth”	85.37%	0.7185
“nose”, “eyebrow”, “ear”	85.37%	0.7976
“face”, “eyebrow”, “ear”	84.15%	0.7843

**Table 6:** The 95% confidence intervals of accuracy and F1-score on the SMIC and SAMM datasets. We use a gray background to distinguish our method from others.

Method	SMIC		SAMM	
	**Acc CI (%)**	**F1 CI**	**Acc CI (%)**	**F1 CI**
SRMCL	[78.31, 79.65]	[0.7770, 0.8004]	[73.91, 75.35]	[0.6472, 0.6726]
LTR3O	[80.42, 81.78]	[0.8069, 0.8195]	[79.45, 80.85]	[0.7469, 0.7679]
ine Off-ApexNet+proto	[73.70, 75.08]	[0.6845, 0.7071]	[71.28, 72.84]	[0.6236, 0.6482]
I3D-MOCO+proto	[76.17, 77.49]	[0.7540, 0.7724]	[70.68, 71.96]	[0.6511, 0.6769]
SRMCL+proto	[78.67, 79.87]	[0.7714, 0.7976]	[74.30, 75.70]	[0.6627, 0.6811]
LTR3O+proto	[82.38, 83.48]	[0.7959, 0.8145]	[80.20, 81.56]	[0.7563, 0.7745]
PCMMT (Ours)	[86.72, 87.66]	[0.8134, 0.8290]	[80.98, 82.26]	[0.8036, 0.8214]

## Data Availability

The datasets used in this study (SAMM, SMIC) are publicly available benchmark datasets and can be obtained from their respective providers. Dataset: SAMM, accessible link: https://helward.mmu.ac.uk/STAFF/M.Yap/dataset.php, accessed date: 17 December 2024; dataset: SMIC, accessible link: https://www.oulu.fi/en/university/faculties-and-units/faculty-information-technology-and-electrical-engineering/center-for-machine-vision-and-signal-analysis, accessed date: 30 December 2024.
